# Epitheloides Angiomyolipom der Niere nach stattgehabtem malignem Melanom

**DOI:** 10.1007/s00120-021-01662-x

**Published:** 2021-10-04

**Authors:** H. Rothe, A. Gaber, B. Dittrich, M. Nagel, M. Tuffaha, B. Hoschke

**Affiliations:** 1 Zentrum für Nephrologie und Stoffwechsel Weißwasser, Albert-Schweitzer-Ring 32, 02943 Weißwasser, Deutschland; 2 Dialysepraxis Leipzig, Käthe-Kollwitz-Straße 16–18, 04109 Leipzig, Deutschland; 3 Carl-Thiem-Klinikum Cottbus, Thiemstraße 111, 03048 Cottbus, Deutschland

**Keywords:** Epitheloides Angiomyolipom, Nierentumor, Melanom, Tuberöse Sklerosekomplexgenmutationen, Zweittumor, Tumorsuppressorgendefekt, Epithelioid angiomyolipoma, Renal tumor, Melanoma, Tuberous sclerosis complex gene mutations, Second primary tumor, Tumor suppressor gene defect

## Abstract

**Hintergrund:**

Die Therapie der epitheloiden Angiomyolipome (eAML) kann eine Herausforderung darstellen, da bei dieser sehr seltenen Unterform der gutartigen mesenchymalen Angiomyolipome anders als bei den klassischen Angiomyolipomen bei bis zu 30 % der Fälle Lymphknotenmetastasen, lokale Rezidive und Fernmetastasen auftreten.

**Ziel der Arbeit:**

Wir berichten hier nach unserer Recherche erstmals in Deutschland von einem Fall von eAML nach stattgehabtem malignem Melanom.

**Material und Methoden:**

Neben der Klinik und Histologie wird die genetische Untersuchung des Tumorgewebes dargestellt.

**Ergebnisse:**

Es fand sich eine somatische, trunkierende Mutation des *TSC2*-Gens („tuberous sclerosis complex“) im Angiomyolipom.

**Schlussfolgerung:**

Die Beziehung zu verwandten Tumorentitäten in der histologischen Diagnostik wird dargestellt und eine mögliche Rolle der genetischen Diagnostik für die Therapieplanung diskutiert.

## Abstract (en)

**Background:**

Therapy of epitheloid angiomyolipomas (eAML) may be challenging, since unlike classical angiomyolipomas this rare subclass of benign mesenchymal angiomyolipomas may present with lymph node metastases, local recurrent disease, and/or systemic metastatic disease in up to 30% of cases.

**Objectives:**

We report here for the first time in Germany a case of eAML after successful treatment of malignant melanoma.

**Materials and methods:**

Clinical and histological findings as well as results of the genetic analysis of the angiomyolipoma are presented.

**Results:**

A somatic, truncating mutation of the TSC2 gene was found in the angiomyolipoma.

**Conclusion:**

The relationship to histologically similar tumor entities are presented and therapeutic options based on the genetic classification are discussed.

## Einführung

Erst vor kurzem, in der Ausgabe vom November 2020 dieses Journals, wurde der Fall eines epitheloiden Angiomyolipoms (eAML) vorgestellt [11]. Handelte es sich dabei um den ersten in Deutschland publizierten Fall eines Lokalrezidivs bei dieser sehr seltenen Unterform der gutartigen mesenchymalen Angiomyolipome, so berichten wir hier erstmals von einem eAML der Niere als Zweittumor nach stattgehabtem malignem Melanom. Im Tumorgewebe wurde eine inaktivierende, somatische Mutation des tuberösen Sklerosekomplexgens („tuberous sclerosis complex“, TSC) nachgewiesen. Die TSC-Gene (1 und 2) sind autosomal dominante Tumorsuppressorgene, deren Genprodukte Hamartin und Tuberin essentielle Regulatoren des „mTOR complex 1“ (mammalian target of rapamycin) ([3]; mTORc1) darstellen.

## Fallbericht

**Figure 1:**
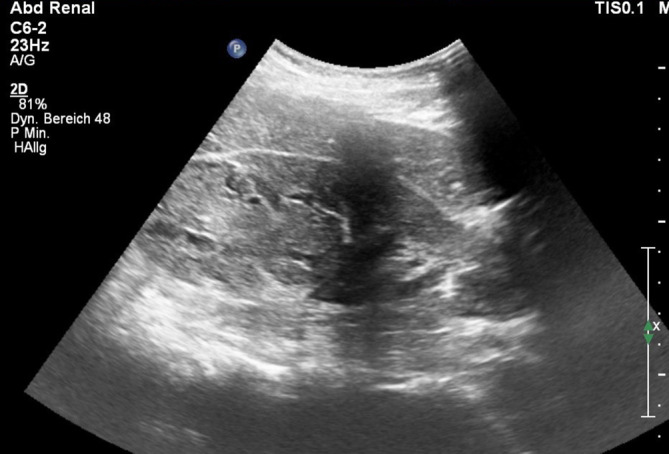

Eine 46-jährige Frau in leicht reduziertem Allgemein- und normalem Ernährungszustand stellte sich wegen rechtsseitiger Flankenschmerzen zur Ultraschalldiagnostik vor. Im März 2018 war ein malignes Melanom vom linken Oberschenkel exzidiert worden. Sonographisch fand sich ein etwa 7 cm großer, echonormaler Tumor am oberen Pol der rechten Niere (Abb. 1).

**Figure 2:**
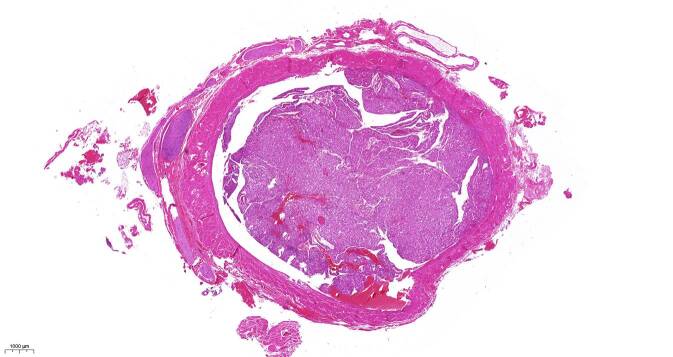

Der zum Zeitpunkt der Diagnostik bereits recht große Tumor hatte durch Infiltration der peripheren Nierenvene eine wandständige Nierenvenenthrombose (Abb. 2) verursacht, wie sich dann erst intraoperativ bei der (aus diesem Grund über einen lumbalen Zugang durchgeführten) Tumornephrektomie zeigte. Diese Nierenvenenthrombose dürfte durch Kapseldehnung wesentlich die lumbalen Schmerzen verstärkt haben, welche zur Diagnosestellung führten.

## Histologische und histogenetische Befunde

**Figure 3:**
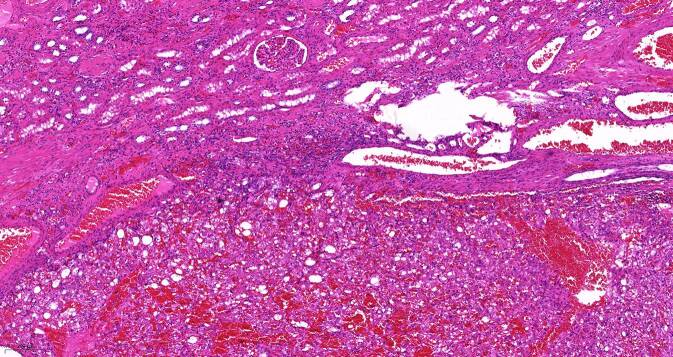

**Figure 4:**
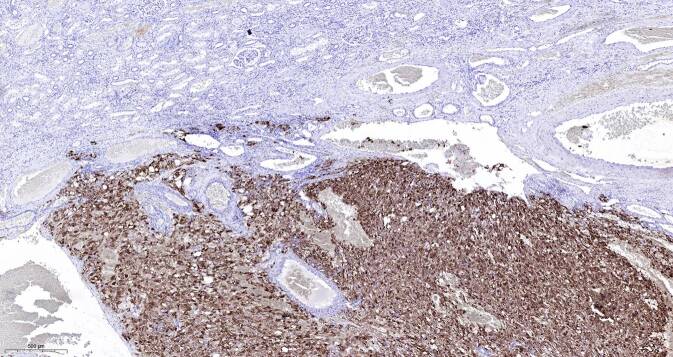

**Figure 5:**
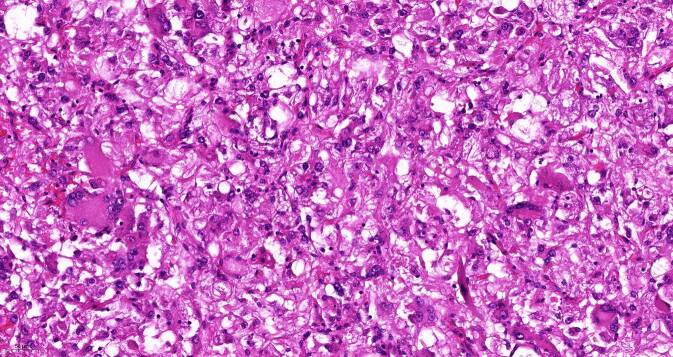

In der pathologischen Aufarbeitung des Präparats fand sich ein 68 mm messendes eAML (Abb. 3) mit Atypien mit sowohl makro- als auch mikroskopischer Hämangioinvasion sowie außer der wandadhärenten Thrombose der V. renalis auch perineuronaler Tumorausdehnung (Tumorformel pT3b V2 R1 cN0 cM0). Immunhistochemisch exprimierten die Tumorzellen den Melanozytenmarker „human melanoma black 45“ (HMB45, Abb. 4) und es fanden sich kleinherdig Abschnitte mit Actin-positiven Muskelfasern (Abb. 5).

In Anbetracht der Vorgeschichte eines (nicht ulzerierten, apigmentierten, superfiziell-spreitenden) Melanoms der Haut im Bereich des linken Oberschenkels 02/2017 wurden ergänzende immunhistochemische Untersuchungen zum sicheren Ausschluss einer metachronen Metastase des malignen Melanoms durchgeführt. Hier zeigte sich eine negative Immunoreaktion der renalen Tumorzellinfiltrate gegenüber den Markern SOX10, S100 und MUM1.

Aus dem Tumorgewebe (Paraffinblock) wurde DNS isoliert (Isolationskit von Qiagen) und nach „massive parallel sequencing“ (Illumina TruSight-One) wurden die Gene TSC1 und 2 analysiert. Dabei fand sich eine somatische Frameshift-Mutation im Exon 4 des TSC2-Gens durch Deletion eines Nukleotids im Codon 130 (NM_000548.4:c.389delA NP_000539.2:*p*.Tyr130Serfs52), welche zu einer weitgehenden Funktionseinbuße des Genprodukts im Tumorgewebe geführt hat.

## Diskussion

### Histologisch verwandte Tumorentitäten

Das Auftreten zweier oder mehrerer Malignome legt den Verdacht auf einen Tumorsuppressorgendefekt nahe, welcher dann gemäß der „Two-hit-Hypothese“ nach Knudson [5] durch eine zweite somatische Mutation im Gewebe zur Ausschaltung des zweiten Allels und damit zur lokalen Tumorentstehung führt. Im vorliegenden Fall kann über die somatische Mutation im Tumor hinaus keine bestimmte Aussage getroffen werden, da mangels Einverständnis der Patientin noch keine Keimbahnuntersuchung vorliegt. Der Melanozytenmarker HMB45, der von den eAML-Tumorzellen exprimiert wurde, gehört mit S100, Melan‑A und Tyrosinase zum „konventionellen Pan-Melanoma-Cocktail“, wobei diese diagnostischen Kriterien ständig weiterentwickelt werden und z. B. erst kürzlich vorgeschlagen wurde, Melan‑A generell durch SOX10 zu ersetzen [1], das auch von uns getestet wurde.

In der Literatur wurden eAML-Fälle mit im Vergleich zu unserer Patientin weit aggressiverem Verlauf beschrieben, wie z. B. der eines 12-jährigen Knaben [4], welcher trotz Behandlung mit Sunitinib, Everolimus und dem Tyrosinkinaseinhibitor Axitinib innerhalb von 4 Monaten verstarb. Obwohl der Tumor die Nierenkapsel nicht durchbrochen hatte, kann das Auftreten eines Lokalrezidivs langfristig auch im vorliegenden Fall nicht ausgeschlossen werden, sodass weitere Therapieoptionen relevant sind.

Neben Angiomyolipomen und Zysten sind von epithelialen Zellen ausgehende Onkozytome die wichtigsten renalen Manifestationen einer TSC-Störung [8]. Die Zellen beim malignen eAML haben Ähnlichkeit mit denen bei der Lymphangioleiomyomatose (LAM; [6]), einer weiteren Folgeerkrankung des TSC: Es finden sich sowohl bei der eAML als auch bei der LAM gutartig erscheinende aber infiltrativ sich ausbreitende spindelförmige oder epitheloide Zellen auf dem Boden mTOR-aktivierender Mutationen in den TSC-Genen, welche Marker von glatten Muskelzellen und Melanozyten exprimieren. Die zu zystischen Veränderungen der Lungen führende LAM befällt fast ausschließlich Frauen nach der Menarche, eine Beschreibung, die auch auf unsere eAML-Patientin zutrifft.

Eine weitere verwandte Klasse von mesenchymalen Tumoren sind die perivaskulären Epitheloidzelltumoren oder PEComas [10], von denen bisher etwa 120 Fälle in der Literatur beschrieben wurden. Auch bei diesem Tumortyp werden die typischen epitheloiden Spindelzellen gefunden, welche Marker sowohl für glatte Muskelzellen als auch Melanozyten exprimieren.

### Marker mit potenzieller therapeutischer Relevanz für etwaige Tumorrezidive

Therapeutische Optionen für etwaige Tumorrezidive und/oder Spätmetastasen sollte man so früh wie möglich klären. Selbstverständlich muss dabei psychologisch behutsam vorgegangen und der beste Zeitpunkt zur Besprechung dieses Problems abgewartet werden, weshalb von unserer Patientin auch noch kein Einverständnis für eine genetische Keimbahnuntersuchung erbeten wurde. Das Vorliegen einer Spätmetastase des Melanoms im Fall unserer Patientin konnte neben SOX10 mit Hilfe eines Antikörpers gegen das S100-Antigen (Rabbit Anti-Cow, Firma DAKO [Dako/Agilent, Santa Clara, CA, USA]) ausgeschlossen werden. Mit dem im Serum nachweisbaren Protein „melanoma inhibiting activity“ (MIA) wurde kürzlich ein Marker vorgestellt [9], welcher in Kombination mit etablierten Markern wie S100B von prognostischer Aussagekraft ist, darüber hinaus aber auch als therapeutisches Target dienen kann.

Auch der Nachweis von TSC1/2-Mutationen beim eAML kann therapeutisch genutzt werden, da die TSC1/2-Genprodukte Hamartin und Tuberin in die mTOR-Signalkaskade eingebunden sind: Die meist gutartigen Angiomyolipome sind überdurchschnittlich häufig mit dem TSC assoziiert, welcher durch Defekte im TSC1- oder TSC2-Gen hervorgerufen wird. Diese im Rahmen der tuberösen Sklerose auftretenden Nierentumoren sind zwar histologisch gutartig, können aber zu schweren Blutungskomplikationen führen: aus unserer eigenen klinischen Praxis wurden 2003 2 Fälle publiziert, welche durch ein akutes Abdomen bei retroperitonealem Hämatom auffällig geworden waren [7]. In beiden Fällen fanden sich TSC2-Mutationen (im Intron 8 des TSC2-Gens bzw. im Exon 14 des TSC2-Gens), welche im Sinne einer Keimbahnmutation aus Tumorgewebe und peripheren Lymphozyten nachweisbar waren. Im vorliegenden Fall konnte wiederum eine TSC2-Mutation aus Tumorgewebe im Sinne einer somatischen Mutation nachgewiesen werden. Ob es sich jedoch um eine (angeborene) Keimbahnmutation handelt, muss mangels Einverständniserklärung zur Untersuchung peripherer Lymphozyten offen bleiben.

Sollte es zu einem Rezidiv oder einer Spätmetastasierung kommen, würde dieser Frage klinische Relevanz bzgl. der Therapieplanung zukommen: Die Rapamycin-Analoga Sirolimus und Everolimus sind schon seit einigen Jahren zur Behandlung mit dem Ziel der Größenreduktion von Angiomyolipomen von der US Food and Drug Administration (FDA) zugelassen [2]. Dabei ist allerdings zu beachten, dass Tumoren auf dem Boden einer TSC2-Mutation bedeutend schlechter ansprechen als solche bei einer TSC1-Mutation. Nach Absetzen der Medikation kommt es meist zu einer erneuten Größenzunahme der Tumoren. Perspektivisch muss bei unserer Patientin in regelmäßigen Nachsorgeuntersuchungen auf etwaige Rezidive geachtet werden, wobei gegebenenfalls auch eine Therapie mit Rapamycin-Analoga nach entsprechender genetischer Untersuchung erwogen werden könnte.

## Fazit für die Praxis

- Wir stellen den Fall einer Patientin mit einem epithelialen Angiomyolipom der Niere vor, welche 3,5 Jahre zuvor bereits an einem malignen Melanom erkrankt gewesen war.
- Sieben Monate nach der Nephrektomie ist die Patientin beschwerdefrei, wird aber in Anbetracht der beschriebenen Lokalrezidive und Fernmetastasen regelmäßig nachuntersucht, wobei auch der genetische Hintergrund einer im Tumor nachgewiesenen somatischen TSC2-Mutation („tuberous sclerosis complex“) gegebenenfalls therapeutisch relevant werden könnte.
